# Is it time for Africa to adopt primaquine in the era of malaria control and elimination?

**DOI:** 10.1186/s41182-022-00408-5

**Published:** 2022-02-25

**Authors:** Richard O. Mwaiswelo, Hamis Kabuga, Eliningaya J. Kweka, Vito Baraka

**Affiliations:** 1 Department of Microbiology, Immunology and Parasitology, Hubert Kairuki Memorial University, P.O Box 65300, Dar es Salaam, Tanzania; 2 Department of Research, Tropical Pesticides Research Institute, P.O Box 3024, Arusha, Tanzania; 3 Department of Medical Parasitology and Entomology, School of Medicine, Catholic University of Health Sciences, P.O. Box 1464, Mwanza, Tanzania; 4 National Institute for Medical Research, Tanga Centre, P.O Box 5004, Tanga, Tanzania

**Keywords:** Malaria, Primaquine, G6PD deficiency, Safety, Control, Elimination, Africa

## Abstract

Primaquine is a gametocytocidal drug known to significantly reduce malaria transmission. However, primaquine induces a dose-dependent acute hemolytic anemia (AHA) in individuals with glucose-6-phosphate dehydrogenase (G6PD) deficiency that has led to a limited use of the drug especially in Africa where the condition is common. The World Health Organization (WHO) now recommends a single low dose (SLD) of primaquine (0.25 mg/kg) as *P. falciparum* gametocytocidal without the need for prior screening of G6PD status. Adoption and implementation of SLD primaquine in Africa may probably reduce malaria transmission, a pre-requisite for malaria elimination. This review therefore, focused on the safety of primaquine for control of malaria in Africa. The literature search was performed using online database Google Scholar, PubMed, HINARI, and Science Direct. Search terms used were “malaria”, “primaquine”, “safety”, “G6PD deficiency”, “large scale” or “mass administration”. Clinical trials in many African countries have shown SLD primaquine to be safe especially in a milder African G6PD A- variant. Likewise, large-scale primaquine administrations outside Africa involving hundreds of thousands to tenths of millions of participants and with severe variants of G6PD deficiency have also shown primaquine to be safe and well-tolerated. Fourteen deaths associated with primaquine have been reported globally over the past 6 decades, but none occurred following the administration of SLD primaquine. Available evidence shows that the WHO-recommended SLD primaquine dose added to effective schizonticides is safe and well-tolerated even in individuals with G6PD deficiency, and therefore, it can be safely used in the African population with the mildest G6PD A- variant.

## Keypoints

- Sub-Saharan Africa contributes about 95% of global malaria cases and related deaths.
- Despite safety concerns adoption of SLD primaquine is needed to further reduce malaria transmission, an essential prerequisite for the elimination of the infection in Africa.
- Large scale administrations of primaquine for control and elimination of malaria have been implemented in other parts of the world where there are severe variants of G6PD deficiency, but only around 1% of the population had mild adverse effects.
- African G6PD A- is a milder variant of deficiency, and the hemolysis that occurs following a single 0.25 mg/kg primaquine administration in this group is usually mild and self-limiting.
- With proper planning and preparation for the management of adverse effects, administration of SLD primaquine plus effective schizonticides, in a form of mass drug administration or seasonal malaria chemoprevention can be used in Africa to reduce malaria transmission.

## Background

Despite recent strides in the control and elimination, malaria infection remains a major global health problem [1, 2]. Malaria is one of the major causes of morbidity and mortality in Sub-Saharan Africa (SSA). In 2020 there were 241 million malaria cases and 627,000 deaths globally, and about 95% of these occurred in SSA [2]. Children under the age of 5 years and pregnant women are the most vulnerable to the infection, with children accounting for two-thirds of the malaria-related deaths [1, 3, 4]. Besides morbidity and mortality, malaria also negatively impacts the African economy [5–8]. Malaria control and elimination in SSA is, therefore, expected not only to alleviate morbidity and mortality but also to improve the economy.

In recent years, malaria elimination has rekindled interest in among stakeholders due to the remarkable strides gained in the past decade of enhanced control. However, the existing tools are still inadequate to eliminate the disease due to asymptomatic careers and lack of effective tools to halt transmission in most endemic areas where the burden is moderate to high. To further reduce malaria transmission and possibly reach the elimination stage, SSA countries need to adopt additional control tools including primaquine, an old but effective tool that has not been fully utilized in the continent. Primaquine offers an additional tool targeting sexual stages of the parasite lifecycle, a critical part of the comprehensive malaria elimination strategy to achieve malaria free target. Despite safety concerns especially in individuals with glucose-6-phosphate dehydrogenase (G6PD) deficiency, which has been a major obstacle for use of primaquine in Africa, the drug has been used successfully in other parts of the world with severe variants of G6PD deficiency, but with no or very minimal adverse effects (AEs) [9]. The World Health Organization now recommends a single 0.25 mg/kg dose of primaquine as *Plasmodium falciparum* gametocytocidal without the need for prior screening of G6PD status [1, 2]. Studies in SSA countries supports the safety of this single low-dose (SLD) even in individuals with G6PD deficiency [10–14]. SSA may therefore, adopt SLD primaquine to reduce malaria transmission and accelerate its elimination in parts of the continent that are already in the pre-elimination stage. This review, therefore, highlights studies on the safety of the WHO-recommended SLD of primaquine for control of malaria in Africa. Emphasis was also given to the safety of previous mass drug administrations (MDAs) of schizontocidal drugs plus either low or higher doses of primaquine, administered either daily or weekly over a prolonged period.

### Search strategy

A literature search was performed to identify publications that reported on primaquine mass administrations or clinical settings, and its safety outcomes, particularly in G6PD deficiency. The search included primary research studies and reviews including systematic reviews and meta-analyses published from 1952 to the present. The literature search was limited to English language publications only, and it was performed using online databases Google Scholar, PubMed, HINARI, and Science Direct. The search keywords included “malaria”, “primaquine”, “safety”, “G6PD deficiency”, “large scale” or “mass administration”. The identified articles were screened manually by titles, and then abstracts to assess eligibility for inclusion based on the predefined criteria. Articles which were not related to the topic of interest were excluded. Eligible articles that reported on primaquine safety, large-scale/mass administration or clinical trial, and G6PD deficiency were identified and included in the review. Studies done in non-human subjects and articles whose full text could not be obtained were excluded.

Validity and quality of all the included studies were assessed based on the following 10 National Institute of Health criteria [15]: population, intervention, comparator, outcomes, time, setting, study design, language, publication type, and publication time frame. The criteria were stated in a form of questions that could be answered “yes” or “no”.

A total of 3521 articles were identified through the databases, and of these, 382 articles related to the topic of interest were screened. Ninety-three full-text articles were assessed for eligibility, and 17 eligible articles were included in the manuscript.

### Global malaria situation and control efforts

In 1900 malaria was endemic to almost all territories of the world [16, 17]. Since then the world malaria map has shrunk following the successful elimination of the infection in Europe, North America, and parts of Asia and North Africa [2, 16]. The gains in the elimination of malaria in these territories were attributed to among other things the use of mass drug administration (MDA) using schizontocidal drugs including chloroquine, and primaquine (PQ) to eliminate *P. vivax* hypnozoites and kill mature *P. falciparum* gametocytes thus blocking transmission, and vector control using dichlorodiphenyltrichloroethane (DDT) [16–19]. No significant gains were made in Africa at the time [16, 20] since the continent was not part of the global malaria eradication campaign of 1955–1969 [20]. Likewise, the emergency and spread of *P. falciparum* resistance against chloroquine reduced the effectiveness of the drug [18]. DDT use was also banned in 1973 due to concerns over its environmental poisoning effect [21, 22]. Conversely, safety concerns against primaquine especially in G6PD deficiency limited its use especially in Africa where the prevalence of the condition is up to 33% [23–25]. As a consequence, currently, Africa accounts for nearly 95% of malaria cases and deaths [2].

The scale-up of malaria control started in the 2000s using tools such as vector control using long-lasting insecticidal nets (LLINs) and indoor residual spraying (IRS), chemoprophylaxis in pregnancy and under-five children, and proper management of clinical cases [26–29] witnessed a 30% reduction in global malaria incidences, and 44% reduction in malaria-related deaths in Africa between 2000 and 2015 [2, 29, 30]. The reduction in malaria incidences has however stalled, and the number of cases increased between 2016 and 2019 [2, 30]. Furthermore, the emergency of corona virus pandemic disrupted the provision of malaria prevention, diagnosis and treatment services in 2020 that led to about 14 million more malaria cases and 47 000 more deaths in 2020 compared to 2019 [2]. Malaria control and elimination efforts have also been weakened by the inherent deficiencies of the available control tools including: low coverage of LLINs and IRS [2, 31]. Furthermore, the spread of *Anopheles* resistance against pyrethroid [20, 31, 32], predominance of outdoor feeding and resting *An. arabiensis* as a consequence of scale-up of LLINs [33], and *Anopheles* behavioral changes including feeding outdoor or at early hours of the evening when most people are not under the bed net protection [33, 34] are hampering the impact of LLINs and IRS. The emergency and spread of artemisinin resistance in parts of Southeast Asia [35–38], and recently in East Africa [39, 40], also threatens the impact of the drug. Artemisinin derivatives are also not potent against mature *P. falciparum* gametocytes, thus cannot prevent the transmission of malaria [41, 42]. Thus, the integration of additional control tools such as primaquine may revamp the control efforts, reduce malaria transmission and accelerate the elimination of malaria in Africa [23, 43, 44].

### Primaquine use, hemolysis, and G6PD deficiency

Primaquine is an 8-aminoquinoline antimalarial drug first synthesized in 1946 [19] and got licensed in 1952 [45]. It has activity against intra-hepatic schizonts and hypnozoites of *P. vivax* and *P. ovale* [45]; thus it is indicated for a radical cure of *P. vivax* and *P. ovale* infection, for causal prophylaxis, and terminal prophylaxis [18, 19, 44, 45]. Primaquine is also potent against mature *P. falciparum* gametocytes, a parasite stage responsible for the transmission of the infection from human to female *Anopheles* mosquito [19, 45, 46]. Whereas it is given daily for 14 days for radical cure of *vivax* and *ovale* malaria, when used as *P. falciparum* gametocytocidal primaquine it is given as a single dose [43]. A single dose of primaquine is sufficient to sterilize mature *P. falciparum* gametocytes, blocking its transmission before it kills and clears them from the circulation [43, 47, 48]. The drug also inhibits the production and maturation of normal sporozoites [47].

Primaquine however induces a dose-dependent hemolysis of the red blood cells (RBCs) in individuals with G6PD deficiency leading to acute hemolytic anemia (AHA) [18, 43, 46, 47, 49–52], an adverse effect that has led to limited use of the drug especially in SSA where the condition is more prevalent [44]. The AHA can be life-threatening since massive intravascular hemolysis with hemoglobinuria may precipitate acute renal failure especially in adults [43, 45]. Besides primaquine dose, the severity of AHA also varies with the clinical status at the time of drug administration, i.e. pre-existing anemia and severity of G6PD deficiency [9, 43, 44]. The risk of hemolysis is markedly high in healthy than in individuals with acute malaria since the latter are often already anemic [43]. Malaria infection itself causes hemolysis with preferential destruction of older RBCs, thus in G6PD deficiency, the proportion of vulnerable older RBCs is lower in malaria than in healthy subjects [9].

The G6PD deficiency is an x-linked anomaly of the RBCs [43, 53]. The anomaly is associated with hemolysis of RBCs in response to certain foods, drugs, infections, or stresses [52–54]. The geographical distribution of G6PD deficiency mirrors that of malaria since the condition provides some protection against the infection [9, 18, 45, 48, 53]. It is more prevalent in Africa, especially among men, with the prevalence ranging from 1 to 33% [18, 19, 44, 45, 48]. Males have only one G6PD allele, thus are either normal or hemizygous deficient, whereas females have two, thus can either be homozygous normal, homozygous deficient, or heterozygous [43, 53]. Heterozygous females have a partial deficiency with some RBCs having normal levels of G6PD whereas others are deficient [43]. Hemolysis is severe in hemizygous males and homozygous females, but its severity in heterozygous varies from that in hemizygous males to that in G6PD normal individuals [43].

There are more than 180 variants of G6PD deficiency [43, 44, 55]. Of the variants, Mediterranean (main variant found in Europe, west and central Asia, and northern India) is the most severe deficiency and is accompanied by severe life-threatening hemolysis on exposure to oxidative agents [38, 39]. The African G6PDA- variant found in SSA is the mildest [44, 48], although severe hemolytic reactions can rarely occur [24, 44]. In non-G6PD deficiency, primaquine is well-tolerated although it can rarely cause non-significant hemolysis [10, 45].

### Previous mass drug administrations of primaquine

Despite the safety concerns, primaquine has over the past 70 years contributed significantly to the global fight against malaria [19, 54, 56–59], especially in low-malaria transmission settings of Asia, the Americas, and Europe [9, 48, 60]. Reports on the large-scale mass drug administration (MDA) of primaquine for the control and elimination of malaria are presented in Table 1. In combination with chloroquine, a 15 mg dose of primaquine was administered for 2 weeks to prevent *P. vivax* relapse in some 250,000 US troops returning from the Korean War between 1950 and 1953 [54, 56–58]. Weekly 45 mg primaquine combined with chloroquine was also administered to the US troops in the Vietnam War [24]. In 1970’s some 28 million people in Jiangsu province, China received primaquine using radical treatment regimens [60]. In Azerbaijan, Tajikistan, Northern Afghanistan, and the Democratic People’s Republic of Korea (DPRK) nearly 8 million people received primaquine-MDA for prevention or elimination *of P. vivax* infection [9]. In Nicaragua, 1.9 million people received a 3-day regimen of primaquine plus chloroquine to control and eliminate *P. vivax* and *P. falciparum* malaria [61]. Likewise, small-scale primaquine-MDAs have been conducted in Malaysia, Cambodia, and Sumatra, Indonesia [62]. Small-scale primaquine MDAs have also been conducted in high malaria transmission-settings of SSA including in Cameroon, Mohel Island, and Tanganyika [62–64]. In all these MDAs, primaquine was deployed without G6PD testing and the prevalence of reported SAEs related to the drug was very low [9, 61–63, 65]. Of note, 14 deaths associated with primaquine have been reported over the past 6 decades, and 12 of them were due to severe hemolysis [24, 44, 66]. One death followed a single 45 mg dose, the rest followed multiple-dose administration [43, 44]. No death is known to have occurred following the administration of SLD of primaquine [43].

**Table 1:** Previous large-scale administrations of primaquine for control of malaria in different parts of the world

Author, country, year	Drug regimen, duration of intervention	Target population G6PD deficiency variant	Target population size (coverage)	Safety
Alving et al. [54], USA, 1950–1953	PQ 15 mg for 14 days Duration: 4 years	Soldiers returning from Korean war G6PD A- variant	250,000	Hemolysis occurred in half a dozen
Kondrashin et al. [9], Afghanistan, 1971–1974	PQ 1971–1973 Duration: 3 years 1973–1974 Duration: 2 years	All individuals (except infants, pregnant, chronically ill) All individuals (except infants, pregnant, chronically ill) Mediterranean variant	1937–14,028 (≥ 90%) 78,000 (not described)	Drug highly tolerated and safe 1% side effects: fatigue, headache, backpain, GIT disorders
Kondrashin et al. [9], Azerbaijan, 1971–1975	PQ 15 mg daily for 14 days Duration: 5 years	All individuals (except infants, pregnant, lactating mothers) Mediterranean variant	10,587–106,555 (87–93%)	≤ 4% had adverse effects ≤ 1% of G6PD deficient subjects had severe adverse effects i.e. red to black urine Hb drop of 3–5 g/dL occurred in G6PD deficient, and 1–2 g/dL in normal subjects
Hsiang et al. [60], Jiangsu, 1973–1983	1973–1976: PQ 30 mg daily for 4 days plus pyrimethamine 50 mg daily. Duration: 4 years 1977–1983: PQ 22.5 mg plus pyrimethamine 12.5 mg daily for 8 days. Duration: 7 years	All individuals in rural areas All index cases from previous year and their contacts	13,389,482–27,974,966 4,446,687–16,534,356	49 G6PD deficiency individuals had acute hemolysis
Garfield et al. [61], Nicaragua, 1981–1982	CQ 350–1500 mg plus PQ 10–45 mg over 3 days Duration: 3 years	All individuals ≥ 1 year	1,900,000 (80%)	Not described
Kondrashin et al. [9], Tajikistan, 1983–1985	PQ (dosage and regimen not described) Duration: 3 years	All individuals except infants, pregnant women, and chronically ill Dushanbe	80,000 (77%)	Side effects were very low (No hard data)
Luo et al., [67], China, 1985–1994	CQ 1500 mg plus PQ 90 mg for 3 consecutive days Duration: 10 years	All individuals	1,052,170 (not described)	Not described
Han et al. [68], Yeom et al. [69], Republic of Korea, 1997–2005	PQ 15 mg/day for 14 days for retired soldiers. CQ 300 mg weekly for active soldiers Duration: 9 years	Active and retired soldiers. (G6PD subjects included)	985,282	Not described
Kondrashin et al. [9], Tajikistan, 1998–1999	PQ (dosage and regimen not described) Duration: 2 years	All individuals Dushanbe	257,200–512,000 (not described)	Not described
Hsiang et al. [60], Jiangsu, 2000–2009	CQ 400 mg daily for 3 days plus PQ 22.5 mg daily for 8 days Duration: 10 years	Index cases of past 1–2 years and all contacts (excluded < 3 years, pregnant, and serious ill G6PD deficient individuals included	1,863,399–1,926,183 (60–98%)	7 subjects, 5 in 2003 and 2 in 2007 experienced hemolysis
Pant et al., [70], DPRK 2002–2010	PQ 15 mg daily for 14 days Duration: 6 years	All individuals ≥ 5 years (except pregnant women and patients with lupus, arthritis, leukemia, hepatitis, or history of hemolysis/hypersensitivity after taking PQ)	378,366–4,904,261 (94–98%)	≤ 4% had adverse effects No cases of severe hemolysis were observed
Deng et al. [71], Comoros Island, 2012	DP plus PQ (dose not described) Duration: 3 months	All individuals except children < 6 months old, pregnant women in 3rd month of conception, patients with liver or kidney disease	97,164 (85.7–93.2%)	153 subjects had adverse effects and were mild. Headache, loss of appetite, dizziness and nausea were the most common adverse effects reported No death or serious adverse effect occurred

### A single low-dose of primaquine for blocking the transmission of *P. falciparum*

In 2010 the World Health Organization (WHO) recommended a single dose of primaquine (0.75 mg/kg equivalent to 45 mg adult dose) for *P. falciparum* malaria transmission-blocking with G6PD deficiency screening [72]. However, concerns related to AHA in G6PD-deficient individuals and the limited availability of G6PD testing in the field hampered the successful implementation of the recommendation [73]. In 2012 the WHO recommended a lower dose of 0.25 mg/kg primaquine for use as gametocytocidal in *falciparum* malaria in low transmission settings without the need for G6PD screening [73, 74]. When coadministered with effective schizontocidal drugs i.e. artemisinin-based combination therapy (ACT), a single 0.25 mg base/kg dose gives maximal transmission-blocking effect [11–13, 75–78], thus accelerating malaria elimination strategy and reducing the rate of emergence of artemisinin-resistant malaria parasites [24]. SLD primaquine has also proven to be well-tolerated and safe in various settings with different G6PD deficiency variants [10–14, 75, 76, 79]. Comparison of hematological changes and prevalence of other adverse events associated with 0.25 mg/kg primaquine administration are presented in Table 2.

**Table 2:** Comparison of hematological changes and prevalence of other adverse events following administration of SLD primaquine

Author, country	G6PD deficiency vs normal	Hb changes and other AEs	Prevalence of AEs	SAE	Treatment required
Gonçalves et al. 2016 [75], Burkina Faso	No	The mean relative percentage Hb drop (− 7.8 g/dL in AL + PQ, − 5.7 g/dL in AL) was more pronounced in individuals receiving 0.25 mg/kg PQ than in those who received AL alone but was not statistically significant. Other observed AEs were mild or moderate and were not different between treatment groups	A total number of subjects with AEs was not reported 18 AEs occurred in the ACT + PQ arm 15 AEs occurred in AL alone	None	None
Mwaiswelo et al. 2016 [10], Tanzania	Yes	Mean absolute Hb drop (−1.48 g/dL in G6PD deficient, − 0.74 in G6PD normal) was statistically significantly different between G6PD status, but relative percentage Hb drop (− 12.6 g/dL in G6PD deficient, − 6.2 in G6PD normal) was not significantly different between G6PD deficient and G6PD normal subjects treated with PQ. The majority of the AEs were mild and self-limiting	42.7% (47/110) in ACT + PQ 40.9% (45/110) in ACT alone	None	None
Bancone et al. 2016 [79], Thailand	Yes	Mean relative Hb drop (− 5.2 g/dL in G6PD deficient, − 3.2 g/dL in G6PD normal) were significantly greater in G6PD deficient than G6PD normal subjects in the PQ arm but normalized during follow-up	A total number of subjects with AEs was not reported Dizziness occurred in 4.8% (79/1659) of subjects		
Dicko et al. 2016 [13], Mali	No	Within person changes in Hb concentration were not significant in any of the treatment groups at any time point	40.0% (6/15) in ACT + PQ 40.0% (6/15) in ACT alone	None	None
Tine et al,. 2017 [11], Senegal	Yes	The mean absolute Hb drop (-1.8 g/dL in G6PD deficient, -1.4 g/dL in G6PD normal) was significantly greater in G6PD deficient than G6PD normal treated with PQ. Only one patient developed moderately severe anemia. Dark urine was more frequent in patients who received PQ. Incidences of AEs were similar in both treatment groups	A total number of subjects with AEs was not reported 205 AEs occurred in the ACT + PQ arm 180 AEs occurred in ACT alone	None	None
Bastiaens et al. 2018 [12], Burkina Faso and The Gambia	Yes	The mean absolute Hb drop was significant in G6PD deficient than in G6PD normal patients in Burkina Faso (− 0.92 g/dL in G6PD deficient, − 0.64 in G6PD normal), but was not significant in The Gambia (− 0.99 g/dL in G6PD deficient, − 1.1 g/dL in G6PD normal). PQ was well tolerated with the majority of observed AEs being mild	43.3% (13/30) in ACT + PQ 40.0% (4/10) in AL alone	None	None
Dicko et al., 2018 [76], Mali	No	Within person change in relative percentage of Hb was not significantly different between treatment arms	65.0% (13/20) in SP + AQ + PQ 55.0% (11/20) in DP alone	None	None
Raman et al. 2019 [14], South Africa	Yes	Mean Hb drop (values not indicated) was more prevalent in G6PD deficient than G6PD normal individuals. Anemia during follow-up was more prevalent in G6PD normal than in G6PD deficient. Other observed AEs were common in both groups, and the majority was mild	31.4% (22/70) in ACT + PQ 26.0% (18/69) in ACT alone	Renal impairment in PQ arm. But the patient failed to disclose a history of renal impairment	None

*AQ* amodiaquine, *AL* artemether-lumefantrine, *ACT* artemisinin-based combination therapy, *DP* dihydroartemisinin-piperaquine, *PQ* primaquine, *SP* sulfadoxine-amodiaquine

## Discussion

Primaquine is the only approved antimalarial drug that can sterilize and kill mature *P. falciparum* gametocytes, and therefore, reduce the transmission of the parasite [19, 45, 46]. However, administered at higher doses of 0.5–0.75 mg/kg, the drug is associated with adverse effects particularly AHA in individuals with G6PD deficiency [18, 43, 46, 47, 49–52]. Recently the WHO recommended a 0.25 mg/kg single-dose primaquine to be added to ACTs for the elimination of malaria in low-transmission settings and in settings threatened by artemisinin resistance without the need for screening of G6PD status [73, 74]. The low dose can also be used in higher transmission settings to reduce the transmission [23]. Despite the evidence showing SLD primaquine to be safe [10–14, 75, 76, 79], many African countries are reluctant to adopt the drug. By 2012, 20 countries worldwide included primaquine as the first-line treatment for *P. falciparum* in their national policy, none was in Africa [23]. Since 2012, several countries in SSA have included SLD-primaquine into policy documents [25], and mainland Tanzania adopted the drug as a treatment policy in 2020 [80], but the actual level of implementation and adherence to these policies is unclear [48]. Integration of primaquine into the malaria control toolbox and as part of the comprehensive elimination strategy may reduce malaria transmission in Africa, a prerequisite for malaria elimination in the continent.

### Safety

The use of primaquine has been limited especially in SSA due to a dose-dependent AHA the drug induces in individuals with G6PD deficiency, a condition occurring in the region at a prevalence of up to 33% [18, 43, 49]. Nonetheless, recent clinical trials conducted in different parts of SSA including Burkina Faso, Kenya, Mali, mainland Tanzania, Senegal, South Africa, and Zanzibar [10–14, 75, 76, 81, 82] have shown that the WHO-recommended SLD primaquine was well tolerated and safe in G6PD A- individuals. Likewise, primaquine MDAs conducted outside Africa including Azerbaijan, China, DPRK, Nicaragua, Republic of Korea and USA [9, 54, 60, 61, 67, 68], involving hundreds of thousands to tenths of millions of participants and with different variants of G6PD deficiency also showed primaquine to be well-tolerated and safe. The drug was safe after its administration at an adult dose of 15 mg daily for 14 days [9, 54, 68], 22.5 mg daily for 8 days [60], 30 mg daily for 4 days [60], 10–45 mg over 3 days [61], or 90 mg for 3 consecutive days [67], either in all individuals [60, 67] or except for children aged below one year [61], or except pregnant women and infants [9], or pregnant women and children aged below 5 years [9], or in adults-only [54, 68]. Importantly, in Azerbaijan and Afghanistan where Mediterranean variant prevalence varies from 0 to 38% and 5 to 10%, respectively, 15 mg primaquine was administered once daily for 14 days [9, 44], and only 1% of the treated individuals experienced transient AEs including dizziness, headache, back pain, dark urine, jaundice, gastrointestinal disturbances, and mild scleral icterus [9]. Similarly, in Tajikistan where Dushanbe is the predominant variant, primaquine was administered with only 1% of treated individuals developing AEs [9]. On the other hand, the African G6PD A- variant is the mildest of all the G6PD deficiency variants [44, 48]; thus it is relatively resistant to primaquine-induced hemolysis [24, 43–45, 66]. In G6PD A-, variant older RBCs succumb first to primaquine-induced hemolysis since they have the lowest content of G6PD, but young reticulocytes replacing hemolyzed RBCs have greater G6PD content, and are considerably more resistant to hemolysis [9, 45, 65, 83]. This phenomenon leads to a mild and self-limiting hemolysis [45, 46, 49]. Therefore, the WHO-recommended SLD primaquine is expected to cause only mild and self-limiting hemolysis in the African G6PD A- variant.

Besides AHA, primaquine is also associated with abdominal pain when taken on an empty stomach [9, 19, 24, 45, 49, 55]. The drug also triggers nausea, vomiting, and mild diarrhea [18, 48]. The severity of the gastrointestinal AEs is related to the dose administered [49]. A 15-mg dose of primaquine given on an empty stomach is associated with only mild abdominal pain, whereas higher doses (30 mg or 45 mg) are associated with mild to severe abdominal discomfort with nausea and vomiting [49]. Taking food before primaquine administration can alleviate the AEs [9, 24, 45, 46]. Food intake also increases the oral availability of primaquine hence improving the drug’s efficacy [19]. On the other hand, primaquine also induces methemoglobinemia, an abnormal accumulation of methemoglobin [19, 49]. Methemoglobinemia is common in individuals with nicotinamide adenine dinucleotide hydrogen methemoglobin reductase enzyme deficiency [9, 19, 24]. This enzyme deficiency is however rare [19]. Methemoglobinemia usually occurs with therapeutic or prophylactic primaquine regimens [19, 45], but very rarely is dangerous [44]; however severe cases may be treated with 300 mg of methylene blue [49]. Neuropsychiatric side-effects such as depression and psychosis [19], hypersensitivity reactions [45], and visual disturbances [49], have also been reported following the intake of primaquine although they are rare. Primaquine is also contraindicated in pregnancy as it increases the risk of intravascular hemolysis to the mother and fetus [18]; however, it is safe for use in breastfeeding women [45, 84].

### Primaquine implementation strategies

Despite the significant role played by primaquine MDAs in the reduction and elimination of malaria in low-transmission settings outside Africa, the same impact may probably not be achieved in SSA in a short time. One major reason is that majority of the SSA countries are still in the malaria control phase [25], thus primaquine implementation strategies that worked in low-transmission settings may probably not work in SSA. Nonetheless, in addition to other malaria control tools the SLD primaquine may probably play a significant role in reducing malaria transmission in SSA, an important prerequisite for elimination of malaria in the region. The SLD primaquine may be implemented using three major strategies namely, i) SLD primaquine plus an effective schizonticides MDA, ii) SLD primaquine added to seasonal malaria chemoprevention (SMC), and iii) addition of SLD to an effective ACT for routine treatment of clinical cases attending the health facilities. The SLD primaquine plus effective schizonticides MDA involving the whole population has potential to substantially reduce malaria transmission and accelerate elimination in SSA. This is because the majority of the individuals in endemic settings of SSA are semi-immune to malaria and carry asymptomatic infection. Due to this, they do not seek the medical attention, whereas relatively few individuals with weak immunity particularly the under-five children and pregnant women are the ones who are likely to develop symptoms and seek medical attention [85–88]. These asymptomatic individuals act as reservoirs of the infection in the population [87, 88]. Therefore, MDA involving the whole population may probably easily capture the asymptomatic individuals and reduce malaria transmission significantly. Previous primaquine MDAs conducted in high transmission settings of Cameroon, Comoros Island, and Tanganyika [62–64] and were able to reduce although failed to interrupt the transmission. However, the reduction of malaria transmission is a prerequisite for elimination of the infection, thus MDA should be implemented despite the failure of the strategy to interrupt the transmission. A significant reduction of malaria transmission in SSA will be an important step in realizing malaria elimination target by 2030. Nonetheless, prolonged primaquine-MDAs in high transmission settings may increase the likelihood of development of parasite resistance against the drug. The SMC is another strategy that may be implemented especially in settings where malaria transmission is highly seasonal [23]. The SMC using sulfadoxine-pyrimethamine plus amodiaquine has been widely implemented in the Sahel region and it has substantially reduced the incidences of malaria infection in under-five children [89–92]. In this region, SLD primaquine can be added to sulfadoxine-pyrimethamine plus amodiaquine to increase the impact of the SMC; however, this will require further investigation to ascertain the safety of the strategy. A similar strategy has been used in China whereby seasonal primaquine plus chloroquine MDA was administered to almost 30 million people and led to a significant reduction of malaria incidences [60]. SMC using ACTs plus primaquine may also be implemented in parts of East and Southern Africa that have highly seasonal malaria transmission. For instance, southern regions of mainland Tanzania have highly seasonal malaria transmission [93], therefore, may use the primaquine plus ACT SMC strategy to further reduce the transmission. Furthermore, some countries including Algeria, Botswana, Cape Verde, Eswatini, Namibia, South Africa, and Zanzibar are nearing malaria elimination stage [25], thus they may use primaquine as a component of SMC to accelerate the elimination of the infection. The SLD primaquine may also be added to ACTs to treat the uncomplicated *P. falciparum* cases detected at the health facilities. This strategy is however useful in settings where malaria prevalence is close to elimination. However, in high transmission settings administration of SLD primaquine to every clinical malaria case cannot have any significant impact since the majority of malaria-infected individuals are asymptomatic [86, 88].

On the other hand, the WHO recommendation is that SLD primaquine should be administered without the need to screen for G6PD deficiency [73, 74]. However, the adoption of SLD policy varies from one country to another, with some African countries such as Mauritania, Mayotte, Sao Tome and Principe, and Cape Verde requiring testing for G6PD deficiency before administering primaquine whereas other countries such as Botswana, Namibia, and Madagascar do not require the testing [25]. The mandatory requirement to test for G6PD deficiency is hampering the efforts to adopt SLD primaquine. Nonetheless, the available evidence on SLD primaquine safety in G6PD deficiency is adequate, therefore, in countries where G6PD deficiency testing is mandatory efforts should be made to remove the restrictions to improve the scale-out of primaquine.

### Limitations

The review had limitations including that only English language articles were included since no one among the authors is fluent in others languages such as French and Spanish, only free databases were used for literature search since the review was not funded, and not all the studies included met all the inclusion criteria.

## Conclusion

Available evidence shows that the WHO-recommended SLD primaquine dose added to effective schizonticides is safe and well-tolerated even in individuals with G6PD deficiency, and therefore, it may safely be used in the African population with the mildest G6PD A- variant. Adoption and roll-out of SLD primaquine in Africa can substantially reduce malaria transmission, an essential prerequisite for the elimination of the infection in the continent.

## Data Availability

Not applicable.

## Abbreviations

ACT: Artemisinin-based combination therapy
AE: Adverse effects
AHA: Acute hemolytic anemia
CQ: Chloroquine
DDT: Dichlorodiphenyltrichloroethane
DPRK: Democratic People’s Republic of Korea
G6PD: Glucose-6-phosphate dehydrogenase
G6PD A-: Glucose-6-phosphate dehydrogenase deficiency African variant
IRS: Indoor residual spraying
LLINs: Long-lasting insecticidal nets
MDA: Mass drug administration
PQ: Primaquine
RBC: Red blood cells
SAE: Serious adverse effects
SLD: Single low-dose
SMC: Seasonal malaria chemoprevention
SSA: Sub-Saharan Africa
USA: United States of America
WHO: World Health Organization
